# Environmental risk assessment for sustainable industrial urban development: The case of northern industrial zone of Pavlodar, Kazakhstan

**DOI:** 10.1371/journal.pone.0320835

**Published:** 2025-04-16

**Authors:** Zhanat Shomanova, Yuriy Nossenko, Meruert Yerkibayeva, Dinara Yessimova, Aikun Kuspanova, Ardak Aldasheva, Kulyash Kaimuldinova, Ruslan Safarov

**Affiliations:** 1 Higher School of Natural Science, Margulan University, Pavlodar, Kazakhstan; 2 Department of Geography and Tourism, Toraighyrov University, Pavlodar, Kazakhstan; 3 Department of Geography and Ecology, Faculty of Natural Sciences and Geography, Abai Kazakh National Pedagogical University, Almaty, Kazakhstan; 4 Department of Tourism and Service Maintenance, Almaty Technological University, Almaty, Kazakhstan; 5 Institute of Natural Sciences and Geography, Abai Kazakh National Pedagogical University, Almaty, Kazakhstan; 6 Department of Chemistry, Faculty of Natural Sciences, L.N. Gumilyov Eurasian National University, Astana, Kazakhstan; CNESTEN: Centre National de l'Energie des Sciences et des Techniques Nucleaires, MOROCCO

## Abstract

This study assesses heavy metal (HM) contamination in soils of an urban industrial zone using statistical and spatial analysis methods. Concentrations of 12 key HMs, including Zn, Pb, Cu, and Ni, were measured using X-ray fluorescence (XRF), with values exceeding background levels several times in certain areas. Pollution indices such as the Pollution Load Index (PLI) and Total Pollution Indicator (Zc) revealed moderate to high contamination levels, with PLI values ranging from 1.05 to 3.38 and Zc values between 0.67 and 51.34. Health risk assessments indicated that the hazard quotients (HQ) exceeded safe thresholds in hotspots, highlighting potential risks. Spatial distribution maps identified industrial activities as the primary source of contamination. Specifically, according to the PLI, approximately 93.757% of the studied area is classified as moderately contaminated, while 0.702% is considered significantly contaminated. These findings provide a baseline for monitoring and mitigating soil pollution in industrial regions while offering insights for sustainable land management.

## Introduction

The processes of industrialization lead to the consolidation of industrial hubs in cities with developed industrial activity [1,2]. A lot of new manufacturing appears around large enterprises [3]. The developed technological infrastructure of industrial hubs determines the location of new large factories on these territories [4]. Such processes, on the one hand, create new jobs, which is important for the social community of the region. On the other hand, the flow of pollution coming from production increases [5,6]. The growth of emissions occurs both due to new plants and factories and due to the increase in production by giant plants [7–10].

An urban industrial zone is an area within a city that is designated for industrial purposes, such as manufacturing, production, and industrial-related services. These zones typically consist of factories, warehouses, and other facilities that focus on industrial activities. They are often strategically located to benefit from existing infrastructure, such as transportation networks, energy supplies, and technological services, which are vital for the operation of industrial enterprises [11].

Assessing the potential environmental risks associated with urban industrial zones is crucial for ensuring the sustainable development of these areas and the well-being of local communities [12]. The combined climate and urbanization pressures on public health and the environment in cities will continue in the coming decades and will increase the necessity to provide more optimal solutions to monitor and assess urban environmental risks [13,14]. Effective environmental risk assessment allows for the identification and evaluation of potential hazards and their potential impacts on the environment, human health, and surrounding communities [15].

Effective monitoring and regulation of toxic elements, particularly heavy metals (HMs), in the environment are crucial for managing these environmental risks. This includes regular assessment of soil, air, and water quality, as well as implementing remediation strategies where necessary to protect the health of ecosystems and human populations. Therefore, this problem is highly relevant and attracts worldwide research attention as it affects environmental quality and human health [16–21].

The assessment of potential environmental risks in an area, particularly those related to pollution, can be performed using various environmental indicators, such as Pollution Load Index (PLI) [22–24] and Total Pollution Indicator (Zc) [25–27]. These indicators can quantify the degree of contamination and help in identifying areas that may require remediation or further investigation.

Pavlodar is a major industrial center in Kazakhstan. It is a multisectoral complex devoted to the production of electricity and fuel-based energy, oil refining, production of construction materials, machine building, and light and food industries [28,29]. Thus, the northern industrial zone (NIZ) of Pavlodar City is a pronounced example of an urbanized and industrial-developed territory [30,31]. Along with the economic positive effects these territories can cause significant changes in the condition of the environment and are supposed to be monitored and studied regularly to assess environmental risks and plan measures for remediation and diminishing of contamination for preserving the well-being of resident people. Recent statistics indicate a noteworthy rise in respiratory diseases and cases of cancer in Pavlodar Region [32–34].

A few studies have investigated the distribution of HMs in Pavlodar city [35–37]. However, most of these publications are dated and fail to account for changes in the industrial areas. There is a lack of literature regarding comprehensive statistical analysis of correlations among element-pollutants and environmental indicators, including PLI, and Zc in industrial areas. Moreover, PLI was not calculated, and its spatial distribution was not studied in Pavlodar region, Kazakhstan. Thus, it is necessary to assess and evaluate the spatial distribution of HMs through a comprehensive monitoring study in an industrial region. The NIZ of Pavlodar City serves as a model region for common technogenic ecosystems and is suspected of contamination. Therefore, this region is a suitable example to investigate.

The aim of this research is to assess the risk level of heavy metal pollution using indices and spatial distribution analysis. This study offers a scientific foundation for assessing the environmental conditions of regions with established industrial production. This is of great importance for maintaining the quality of life and promoting sustainable development in regions with rapidly expanding industries.

## Methods

### Research area

The focus of the research is the NIZ located in Pavlodar city. Pavlodar is the administrative center of the Pavlodar District in the northeastern region of the Republic of Kazakhstan. Pavlodar is one of the oldest and most beautiful cities of the Republic located on the coast of Irtysh - the largest river in Kazakhstan.

The largest representatives of the metallurgical industry in the Pavlodar District are the Pavlodar aluminum plant, the Kazakhstan electrolysis plant, and the Aksu ferroalloy plant [38]. The mining industry includes such large enterprises as coal mining companies “Bogatyr Akses Komir”, and “Maicuben West”. In particular, in the NIZ of Pavlodar city one of the largest Kazakhstani manufacturers of petroleum the Pavlodar petrochemical factory placed, as well as combined heat and power plants TPP-2, TPP-3, cardboard and roofing rubber plant, caustic soda production plant “Kaustik”.

The city of Pavlodar is located in the chestnut soil belt, where soils are predominantly uniform. These soils have formed under arid climatic conditions and dry steppe vegetation. The vegetation cover is represented by associations of feather grass and fescue with the presence of sand sedge, steppe timothy grass, and Artemisia frigida [39].

The soil-forming material consists of unsalted ancient alluvial sandy loams. Groundwater is predominantly fresh or slightly mineralized, lying at depths of 2.72–8.12 meters, and does not significantly influence soil formation processes [40]. Deep effervescent chestnut soils are widely distributed across the study area [41]. The parent materials, by genesis and granulometric composition, fall into several groups. Up to depths of 1.5–2.0 meters, ancient alluvial sands, sandy loams, and light loams predominate, underlain by sand-gravel deposits. Deeper layers consist of Neogene clays serving as regional aquicludes [40]. The region is open to the influence of air masses from the Arctic, temperate and southern latitudes, under the influence of which a type of continental climate is formed here, which is characterized by the aridity of the spring-summer period, long and cold winter (5-5.5 months), hot and short summer (3 months). Insufficient and unstable annual precipitation with their summer maximum and significant wind activity throughout the year.

The territory of the NIZ lies in the dry subzone of the steppe zone, the total solar radiation varies from 95 to 110 kcal/cm^2^ per year. The average annual precipitation ranges from 200 to 310 mm. The average annual air temperature is positive and is 1-2 °C.

The duration of the warm period with an air temperature above 0 °C is on average 190 days. The duration of the period with an average temperature above 10 °C is 140 days with the sum of temperatures during this time being 2400 ° C. In most cases, the first autumn frosts are observed in the middle and end of September, and the last spring frosts are in the middle and end of May. The frost-free period lasts on average 120 days from May to September inclusive.

In some very severe winters, the air temperature drops to minus 45-49 °C (absolute minimum), and the summer absolute maximum is 40-43 °C of heat. The absolute annual amplitude of air temperatures is 88-90 °C. The winter period usually begins in the second half of October and lasts until April 15-20. Winter months are characterized by great instability of air temperature, in some years significant deviations from the norm by 8-11 °C to one side or the other are possible. Along with severe frosts, thaws with air temperature rise to + 5, + 6 °С are possible in winter.

Snow cover appears in the last decade of October, in some places in early November. A stable snow cover forms around November 10-15, which lasts in northern areas until April 5-10. The number of days with snow cover is 130-155, with its height rarely exceeding 20–25 cm. In wintertime, ice is often observed, negatively affecting vegetation. The snow and ice covering, combined with severe frosts, causes deep soil freezing up to 2–3 meters [39].

The highest wind speeds are observed in spring. The windiest months of the year are March, April, May, and sometimes June. The average monthly number of days with strong winds of more than 15 m/s per year is 35 days. The probability of calm days per year is 5-6%. The average annual wind speed is 4-5 m/s.

The combination of air dryness with high wind speeds and low precipitation causes severe drying of the upper soil layer, dust, and sandstorms. They have the greatest impact on vegetation in May, as the soil surface is practically open, loosened, and the top layer dries out quickly in the absence of precipitation and easily succumbs to deflation.

Thus, the climate of the study area is characterized by the following features:

1) a long and severe winter with little snow cover and strong winds;
2) hot, dry, windy summer, accompanied by frequent dry weather;
3) an abrupt transition from winter to summer, rapid snow melt, when moisture does not have time to penetrate the ground;
4) insufficient precipitation, unfavorable distribution of precipitation during the year;
5) dry and cold autumn;
6) winds blow throughout the year and are especially strong in spring and the first half of summer.

Due to the presence of a wide range of soil-forming conditions, the soil cover is characterized by great diversity. Soil-forming rocks are sands and sandy loam, less often - loam, so the soil has a light mechanical composition.

### Sampling and analysis methods

**Fig 1:**
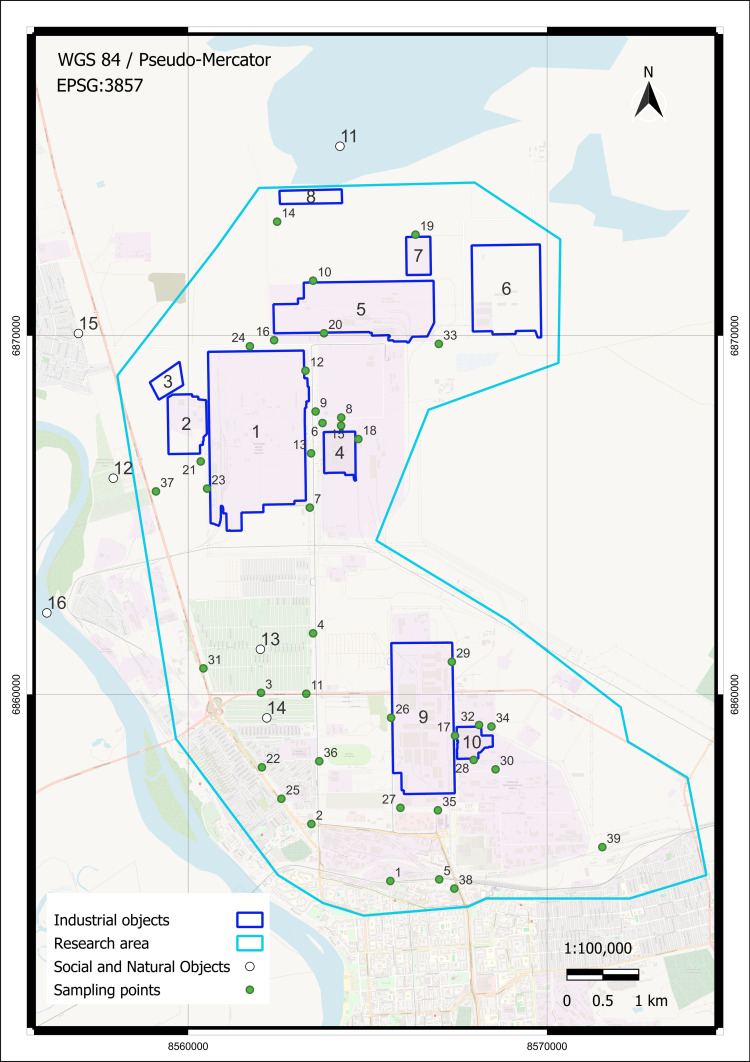
Pavlodar northern industrial zone research area (202.29 km^2^) with sampling locations. Industrial, social, and natural objects: 1—Pavlodar petrochemical plant, 2—Neftechim LTD, 3—solid waste accumulator, 4—TPP-3, 5—former Pavlodar chemical plant (industrial site 1), 6—former Pavlodar chemical plant (industrial site 2), 7—calcined petroleum coke production plant, 8—ponds for mercury waste, 9—KSP-Steel (former Pavlodar tractor plant), 10—TPP-2, 11—Balkyldak lake, 12, 13, 14—residents’ summer houses (dachas), 15—Pavlodarskoye Village, and 16—Irtysh River. 1-39 (green circle mark) — sampling points locations. Basemap: Esri OSM Vector Basemap (CC BY 4.0), Data: OpenStreetMap contributors.

Soil samples were collected from September 20 to October 5, 2022, at 39 locations in the study area. The sampling map is shown in Fig 1. Sampling sites were chosen in areas with minimal vegetation, avoiding grass and bushes, to access open soil surfaces and reduce potential interference from organic material. Preference was given to sandy soils, avoiding rocky substrates or any random debris or garbage present on the ground, to maintain consistency in the type of material analyzed. Additionally, sampling locations were distributed systematically across the entire research area to ensure comprehensive spatial coverage, with particular attention given to sites close to major industrial facilities.

After removal of the humus layer, 5 soil samples weighing ~ 200 g were collected at each site using a hand shovel from a depth of 0-20 cm excluding the top-layer of ~ 2 cm. Samples from each site were mixed to obtain a total of 39 pooled samples with an estimated weight of 1 kg. Samples were transported and stored in plastic containers to protect them from direct sunlight.

Sample preparation involved air-drying all samples, sieving them through a 2-mm sieve, and grinding them into fine powders (200 mesh) using an agate mortar. The powdered samples were stored in plastic containers at room temperature until analysis. For pellet preparation, the powders were mixed with wax as a binder in a 1:9 ratio and pressed onto a boric acid substrate that had been pre-pressed at 5 tons. The final pressing was performed at a pressure of 15-20 tons to create stable pellets suitable for XRF analysis.

The X-ray fluorescence (XRF) analysis was conducted using the Axios PANalytical wavelength-dispersive spectrometer (Axios, Malvern Panalytical, United Kingdom) equipped with a 1 kW Rh-tube, operating under vacuum conditions. The instrument features two detectors—flow and scintillation—and is capable of determining elements ranging from Na to U. The detection sensitivity is approximately 0.01% for light elements and 0.003% for heavy elements, with sample rotation included as part of the measurement process.

For soil samples, the semi-quantitative analysis software package “Omnian”, developed by PANalytical using the fundamental parameters method and a set of standard reference materials, was utilized. The relative error of this method is approximately 10%. The total measurement time per sample, including loading and unloading, was 25 minutes.

The spectrometer’s calibration and verification were performed by an accredited metrology institution following a standardized methodology. A certified reference material (CRM) of lead concentrate composition was used for verification. The spectrometer is certified as a working measuring instrument with a class accuracy of ± 0.5%.

### Environmental risk indicators calculation

In this study, an assessment of potential environmental risk was performed by studying soil contamination levels by the quantification of the complex of conventional indicators such as contamination factor (CF) and the PLI, hazard quotients (HQ), Zc, and integral assessment score (B). These indicators are valuable tools for evaluating the level of trace-element contamination in soils. They also enable us to compare the results with other areas that may be potentially polluted.

The CF was calculated as the ratio between the HM concentrations (mg kg^-1^) in every soil sample and the background values taken from the environmentally none-affected territory near Pavlodar City [42].


$${\text{CF = C}}_{\text{Soil}}{\text{/ C}}_{\text{Background}}\text{,}$$ (1)

CF values were calculated for HMs with background values: Cr, Mn, Fe, Zn, Sr, Cu, Pb, and Ni. As well V, Co, and Hg were presented in some samples in low concentrations, but generally, the concentrations were lower than the analytical detection values.

The PLI expresses contributions of contamination from each HM and was calculated for every point for obtaining the PLI distribution map as well as a complex PLI value integrating the whole dataset for the research area. PLI was calculated as the *n*th root of the number of multiplied CF values in accordance with the following formula [22]:


$$\text{PLI = }{\left({\text{CF}}_{\text{i}}{\text{× CF}}_{\text{j}}{\text{× ··· × CF}}_{\text{n}}\right)}^{\text{1/n}}\text{,}$$ (2)

In samples with one or more HMs below the analytical detection limit, the *n* value was taken as the amount of the HMs with detectable concentrations. Also, when CF_i_ ≤  0.7, this HM was not taken into account. Levels of contamination followed the distribution were published by Wang et al. [20].

Risk characterization for this study was quantified by the HQ which is the ratio of the potential exposure to a selected metal, relative to the level at which no adverse effects are expected [43].


$$\text{HQ = Exposure Concentration/ Reference Concentration }\left(\text{RfC}\right)\text{,}$$ (3)

where exposure concentration is the amount of HM in the soil at a given point (mg kg^-1^). RfC is a maximum permissible concentration (MPC), or a permissible value of polluting chemical compound contained in soil, water, or air that does not affect - directly or indirectly - the living organisms. For soils, it is conventional to use mg kg^-1^ units. A HQ ≤  1 indicates that adverse effects are not likely to occur, and thus can be considered to have negligible hazard. If the HQ >  1, the adverse health effects are possible. MPC of toxic elements including HM are established in Hygienic standards [44].

To characterize the level of pollution of the landscape profile the total pollution index Zc was used, which is defined as the sum of the contamination factors of the individual components of pollution according to the formula:


$${\text{Zc = CF}}_{\text{i}}{\text{+ CF}}_{\text{j}}\text{+}...{\text{+ CF}}_{\text{n}}\text{– }\left(\text{n – 1}\right)\text{,}$$ (4)

where *n* is the number of abnormal elements, where CF ≥  1.5.

Zc indicator characterizes the degree of chemical contamination of soil and ground of the studied area with harmful substances of different natures including HM. Values of the indicator of chemical pollution and the corresponding categories of soil contamination were published by Urazmetov et al. [45].

CF and Zc are criteria that characterize the degree of evolution of geochemical risks at a particular site. Analysis of geochemical risks allows you to determine the level of accumulated geoecological damage and assess the level of pollution.

Comparisons can be made in a more flexible and meaningful way with the versatile integral index. One of the integral indicators that takes into account differences in the toxicity of elements is an integral assessment score based on total concentration factors [26,46].

The integral assessment score is derived from the prescribed equation (5).


$$B=\frac{D_{f}\times 100}{D},$$ (5)

where b – integral assessment score from 0 to 100, D – total concentration factor accounting toxicity of elements (6), and Df – total concentration factor for the background soil (assessment score - 100) (7).


$$D=\sum CF\times K_{i},$$ (6)

where CF – contamination factor (1), K_i_ – coefficient of significance of HMs, which is inversely proportional to the MPC (1/MPC). When the concentration of a given HM does not exceed the value of MPC, it is assumed to be equal to MPC and only the excess elements are taken into account [26].


$$D_{f}=\sum K_{i}$$ (7)

Therefore, as the value of B gets closer to 100, the state of the soil in the area under investigation is more toxicologically neutral.

### Statistical analysis and visualization

The quantitative data were processed utilizing MS Excel 2021 [47], QGIS 3.28.6 [48], and Python scripts [49]. Basic descriptive statistical analysis of the sample data was performed in MS Excel to obtain the maximum, minimum, mean, standard deviation, and coefficient of variation for the sampled HM content.

The QGIS software was utilized for spatial visualization, and the SAGA (ver. 7.8.2) module’s multilevel b-spline function [50] was applied to analyze the spatial distribution characteristics of HMs in soils on the studied territory. The resulting raster grids were subsequently employed to create polygons of isolines utilizing the “Contour Lines from Raster” function of the SAGA module.

The data obtained underwent multivariate statistical analysis, including correlation analysis and principal component analysis (PCA). Statistical investigation was conducted using Python script, employing functions “StandardScaler” and “PCA” from the Sklearn library [51], and function “corr” from the Pandas library [52]. Graphical visualization of the statistical data analysis results, such as plots and heatmaps, was performed using Seaborn [53] and Matplotlib [54] libraries.

## Results

### Geochemical characteristics and spatial distribution of HMs in soil

Total HM concentrations obtained from surficial soil samples are presented in S1 Table. The most prevalent elements are Fe, Mn, and Cr with average concentrations of 20406.92, 553.85, and 203.85 (mg·kg^-1^) correspondingly. Also, concentrations of Sr and Zn were high and reached 173.33 and 121.28 (mg·kg^-1^). Co, Hg, and Mo were detected in the single samples. V was detected in 4 locations. According to the value of the average contents, the studied elements were arranged in the following descending order:


Fe> Mn> Cr> Sr> Zn> Cu> Pb> Ni> Mo > V> Hg> Co.

According to the general toxicological GOST (State Norms and Standards) 17.4.1.02-8 [55], detected HMs Pb, Hg, and Zn relate to highly hazardous elements. Mo, Cu, Co, Ni, and Cr are moderately hazardous. Sr, Mn, and V are low hazardous. Analogous distribution by toxicity was supported by many studies. So in [56] was revealed that pollution index values decreased in the order


Hg> Cd> As> Cu> Pb> Zn> Ni> Cr.

The statistical information on the data of elemental content in soil samples is shown in Table 1. Concentrations of HMs in soil were compared to MPC given in State hygienic standards used in the Republic of Kazakhstan (as well as in Russia and other CIS countries). MPCs for gross forms of metals not established in standards were taken from reliable scientific and technical sources. The used values of MPC are presented in Table 1.

**Table 1:** Statistics on HM concentrations in the northern industrial zone of Pavlodar.

Element	Minimum (mg·kg^ − 1^)	Maximum (mg·kg^ − 1^)	Mean (mg·kg^ − 1^)	Median (mg·kg^ − 1^)	Standard Deviation (mg·kg^ − 1^)	Variation Coefficient, %	MPC (mg·kg^ − 1^) ^1^	Sample over MPC, %
Fe	11,380	34,360	20,406.92	19,860	4810.28	24	40,000 ^3^	0
Mn	240	1900	553.85	460	366.24	66	1500	8
Cr	0	820	203.85	150	206.88	101	200 ^4^	79
Sr	110	250	173.33	170	23.24	13	600 ^5^	0
Zn	30	910	121.28	70	159.10	131	55	79
Cu	0	630	64.87	0	110.06	170	33	46
Pb ^2^	0	200	46.92	50	42.56	91	32	67
Ni ^2^	0	150	34.10	0	47.65	140	20	36
Mo ^2^	0	440	11.28	0	69.55	617	50 ^6^	3
V ^2^	0	90	7.69	0	23.26	302	150	0
Hg ^2^	0	100	2.56	0	15.81	618	2.1	3
Co ^2^	0	80	2.05	0	12.64	617	50 ^7^	3

^1^MPCs were used for gross forms of metals in soil recommended by hygienic standards in [44,57] as well in [58].

^2^Element was not detected in background soil samples. The MPC value was used for calculations.

^3^MPC was not established. Approximately permissible concentration (APC) value was used for the study according to [59], ^4^ [60], ^5^ [61,62], ^6^ [63], and ^7^ [64].

The average values of iron, strontium, and vanadium did not exceed the limit of MPC. The percentage over MPC for Cr, and Zn was 79%; for Pb, Cu, Ni, and Mn - 79%, 67%, 46%, 36%, and 8% respectively; for Mo, Hg, and Co - 3%. The content of HMs in different points varies significantly, for example, the concentration range for Mn is 240-1900 mg·kg^-1^, Cu – 0-630 mg·kg^-1^, Zn – 30-910 mg·kg^-1^. The lowest ranges of concentrations refer to Sr and Fe with corresponding variation coefficients of 13% and 24% respectively.

The variation coefficient reflects the spatial variation degree of elemental content at different locations. If this is less than 33%, the spatial distribution is uniform, in the range of 33-64% distribution is random when more – distribution is clustered [65]. Thus, the distribution of Sr and Fe is uniform, and these elements are not considered toxic pollutants by the level of content. The other detected HMs are characterized by a cluster type of spatial distribution with variation coefficients from 66 for Mn to 618 for Hg. Content variations of Mo, Hg, and Co at different sampling locations showed an anomalous distribution with variation coefficients 617-618%. In the case of Mo, Hg, and Co, the soil samples presented a very low content. Out of the 39 soil samples collected, 38 exhibited metal concentrations below the analytical detection limit.

**Fig 2:**
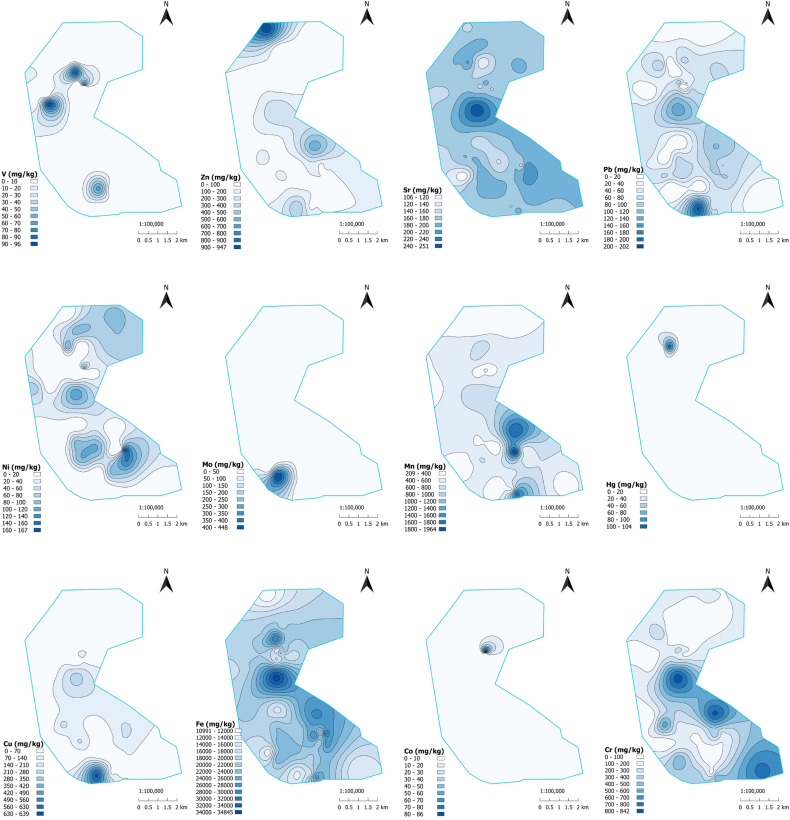
Distribution of heavy metal concentrations in the studied area.

The maps of the spatial distribution of detected HMs on the studied territory are presented in Fig 2. The observed heterogeneity of HM content in soil samples is characteristic of technogenic industrial areas. In these areas, the placement of industrial wastes in heterogeneous dumps and the dispersion of particles into the environment as a result of industrial activities, meteorological phenomena, and leaching into subsurface soil layers usually create the distribution of pollutants without visible structure and clear trend.

**Fig 3:**
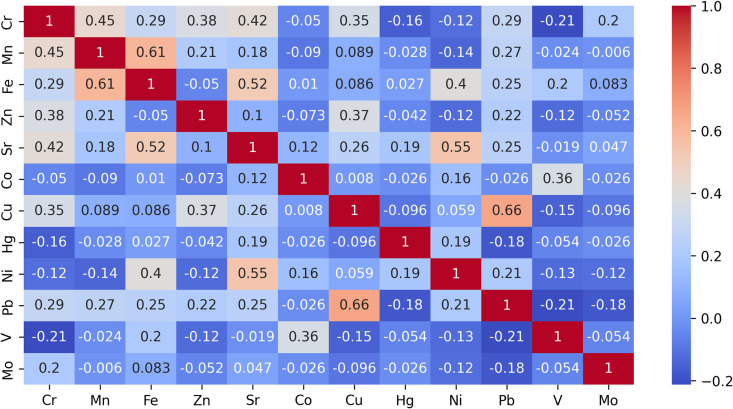
Pearson correlation between HM concentrations in the study region.

To evaluate correlations between different elements in the study area, we utilized a correlation matrix with Pearson’s coefficients. The Pearson correlation coefficient indicates the level of linear association between two variables on a scale from -1 to 1. A score of -1 indicates a fully negative linear correlation, while 0 signifies a lack of linear correlation, and 1 represents a complete positive linear correlation. Fig 3 presents the correlation matrix computed from the HM elemental composition data in the study region.

From the correlation matrix, we can see patterns and trends in the data. The Pearson’s coefficient (r) for the following pairs shows a moderate positive correlation: Pb-Cu (+0.66), Fe-Mn (+0.61), Sr-Ni (+0.55), and Sr-Fe (+0.52). As the concentration of one element increases, so does the concentration of the other; however, there are also exceptions and variations. These HMs may share common factors that influence their distribution in the environment, but there are also some differences. The lowest r values do not fall below -0.21, indicating that no significant negative correlation exists between certain elements. As a result, it can be inferred that these HMs do not exert opposing effects or influences on one another in the environment.

There is no linear relationship between Mo and Hg and any other components, as indicated by the low or close to zero correlation coefficients. This suggests that the concentration of these elements does not consistently vary with any other HM. These two metals may have unique sources or pathways of contamination in the environment.

For pairs with the highest Pearson’s coefficient, including Pb-Cu, Fe-Mn, Sr-Ni, Sr-Fe, and Fe-Ni, the calculated values of the t-statistic for a paired t-test (two-tailed test) were obtained. These values were compared with the critical value determined by the Student’s table.

Comparing the t-statistic with the critical value from the Student’s table is a method for testing hypotheses using the t-distribution. The t-distribution describes the behavior of the test statistic t, which measures the difference between two sample means or between a sample mean and a population mean. The degrees of freedom, which are the number of independent observations in the sample minus the number of estimated parameters, affect the t-distribution. In the case of 39 sampling locations, the degrees of freedom (df) are 37.

The study formulated both null and alternative hypotheses. The null hypothesis asserts that there is no significant difference or relationship between the observed elements. Conversely, the alternative hypothesis claims the opposite of the null hypothesis. The significance level (alpha) indicates the probability of rejecting the null hypothesis when it is true. Typically, alpha values of 0.05, 0.01, or 0.001 indicate the desired confidence level of the results. The chosen significance level for the test is 0.01, indicating a 1% probability of rejecting the null hypothesis when it is true. Lower alpha values increase the stringency of rejecting the null hypothesis, reducing the likelihood of committing a type I error. Nevertheless, this also raises the likelihood of making a type II error, which means that there may be a true effect or relationship missed.

The critical t-value was obtained from the Student’s table. This table provides critical t-values for various degrees of freedom and significance levels. According to the Student’s table at n-2 =  37 and α =  0.01, the critical value is 2.715. This indicates that if the t-statistic modulo is above 2.715, the mean values of the two samples are statistically significant at the 0.01 level. If the t-statistic modulo is less than 2.715, then the difference between the means of the two samples is not statistically significant at the 0.01 level. Conversely, if the t-statistic is greater than the critical value, we can reject the null hypothesis and conclude that the results are statistically significant. If the t-statistic is less than or equal to the critical value, we cannot reject the null hypothesis and must conclude that the results are not statistically significant.

We have also computed the p-values for these pairs. The p-value represents the likelihood of obtaining a t-statistic that is as extreme or more extreme than the one that was obtained from the data, assuming that the null hypothesis is true. The p-value can be useful for analysis by comparing it with a significance level, which acts as a threshold to determine whether the null hypothesis should be rejected or not. If the calculated p-value is less than or equal to the pre-determined significance level, we can reject the null hypothesis and determine that there is indeed a statistically significant difference between the means of the two analyzed features. Conversely, if the p-value is greater than the significance level, we are unable to reject the null hypothesis and must conclude that there is not enough evidence to support a difference between the means of the two features (Table 2).

**Table 2:** Average environmental impact indicators in the NIZ of Pavlodar.

HM pair	Pearson’s coefficient (r)	t-statistic	p-value	degrees of freedom (df)	significance level (α)	t-critical value
Pb-Cu	0.66	-0.937	0.35	37	0.01	2.715
Fe-Mn	0.61	25.368	7.69			
Sr-Ni	0.55	16.189	2.40			
Sr-Fe	0.52	-25.929	1.73			
Fe-Ni	0.40	26.106	1.08			

**Fig 4:**
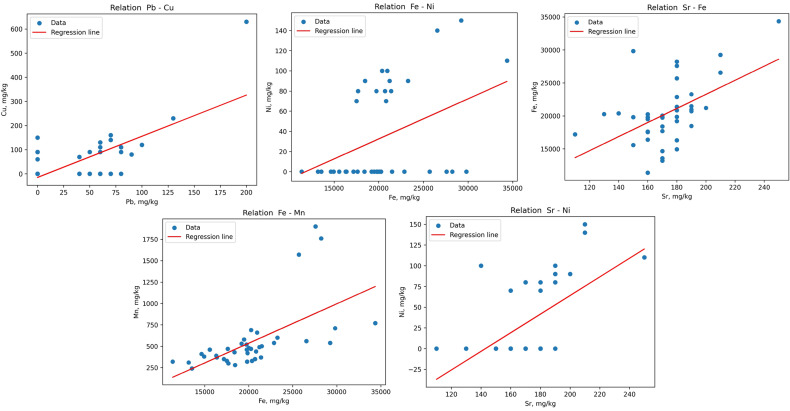
Scatter plots and regression lines for heavy metal pairwise correlations in soil samples.

We utilized a scatter plot, a graph that displays the values of two variables as dots on a coordinate plane, to visually illustrate Pearson’s coefficients. By drawing a line of best fit, a straight line that minimizes the distance between the dots and the line, we can determine the slope and direction of the line, which directly correlate to Pearson’s coefficient of the two variables. The scatter plots for the analyzed pairs of elements are displayed in Fig 4.

The statistical significance of correlations in pairs Fe-Mn, Sr-Ni, Sr-Fe, and Fe-Ni is evident. Fe-Mn exhibits the strongest correlation, suggesting similar sources of environmental contamination for these metals. The scatter plots for Sr-Ni and Fe-Ni pairs show variations in the accumulation of these HMs in different locations. Ni was not detectable in many points, whereas Fe was detected in every sample. Excluding the samples where Ni was not detected, we found statistically significant and strong positive correlations between Fe and Sr. Additionally, we observed a statistically significant moderately positive correlation between Sr and Fe, which suggests a similar or accompanying nature of their entry and distribution in the soil.

However, the calculated p-values were higher than the estimated significance level, indicating that the data lacks the strength to reject the null hypothesis. Therefore, there is insufficient evidence to assert that the relationships are completely clear. This is a common occurrence due to the challenging technogenic nature of introducing these elements into the environment. Emissions from multiple enterprises and varying forms of contaminants overlapping each other create a complicated landscape of the spatial distribution of the elements-pollutants.

Meanwhile, a p-value exceeding the significance level does not equate to acceptance of the null hypothesis or its veracity. It merely signifies that the available data is inadequate for rejection or support of the alternate hypothesis. Other factors may affect the results, such as sample size, measurement error, confounding variables, or chance.

**Fig 5:**
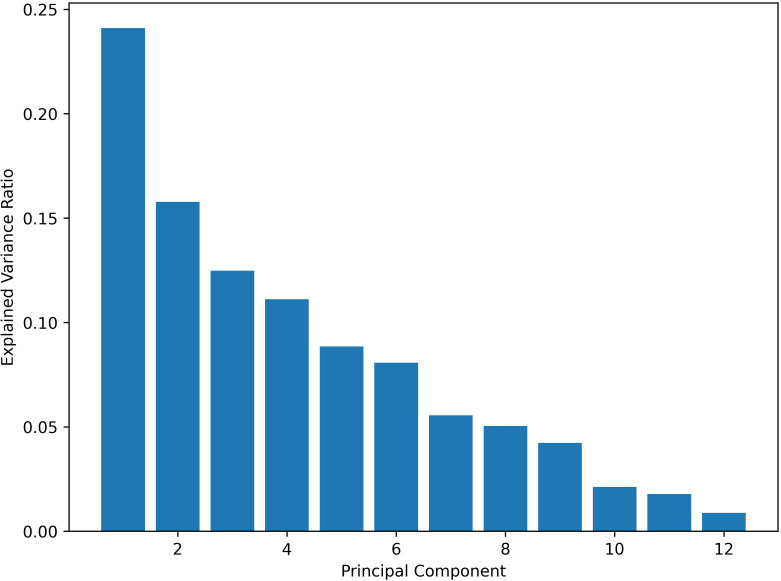
Scree plot of principal components.

Principal component analysis (PCA) is a statistical method used to decrease data set complexity by transforming numerous interrelated variables into fewer, uncorrelated variables known as principal components (PCs). In our study area, we applied PCA to the soil HM concentration dataset. By effectively reducing the original dataset consisting of 12 HM variables, PCA has resulted in a smaller set of principal components. The scree plot obtained is displayed in Fig 5. We preserved the initial 8 principal components, which jointly account for around 90% of the overall variance in the data. This dimensionality reduction simplifies data interpretation while retaining the majority of relevant information.

**Fig 6:**
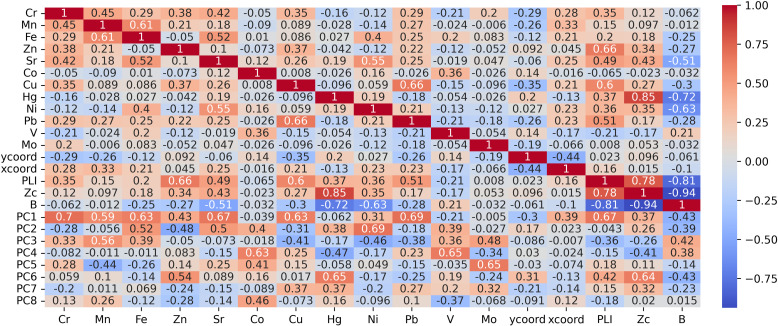
Correlation matrix for HMs, coordinates, environmental indices, and principal components.

The PC combinations obtained were added to the full matrix containing sampling coordinates, HM concentrations, and environmental indices to perform a correlation analysis. This enables us to estimate the elements that have a higher influence on environmental indicators and have the greatest contribution to each PC. Afterward, we can visualize the PC scores on a spatial graph reflecting the distribution of pollutant groups. Fig 6 represents the results of the correlation analysis for the PCA data.

Analyzing the loadings of each HM on the principal components provides insight into their contributions to the extracted factors. Within PC1 Cr, Pb, Sr, Cu, Fe, and Mn exhibit the highest positive loadings, indicating a common source or contamination pattern for these metals. Among the other principal components, PC1 has the highest correlation with PLI (moderate positive), with less emphasis on the correlation with Zc and B. PC2 exhibits a moderate negative correlation with Ni. PC3 exhibits a moderate positive correlation with Mn, PC4 displays a moderate positive correlation with Co and V, and PC5 is emphasized by the load of Mo. PC6 is mainly influenced by Zn and Hg, and this component accurately reflects the Zc. PC7 and PC8 do not exhibit any significant differences or positive correlations with any HMs, and therefore, their meanings are statistically insignificant.

Thus, it can be inferred that the PLI was primarily impacted by a broad range of metals including Cr, Pb, Sr, Cu, Fe, and Mn. In contrast, Zc more precisely indicates the presence of highly toxic elements like Zn and Hg. Meanwhile, the B uniformly represents the average concentration of all metals with no significant dependencies or meaningful differences observed. When comparing correlations among environmental indices, it is evident that Zc and PLI exhibit a strong positive correlation (0.78), while showing a strong negative correlation with B (-0.81 and -0.94). This is logical since higher B scores reflect a better environmental situation, which contradicts the Zc and PLI indices.

**Fig 7:**
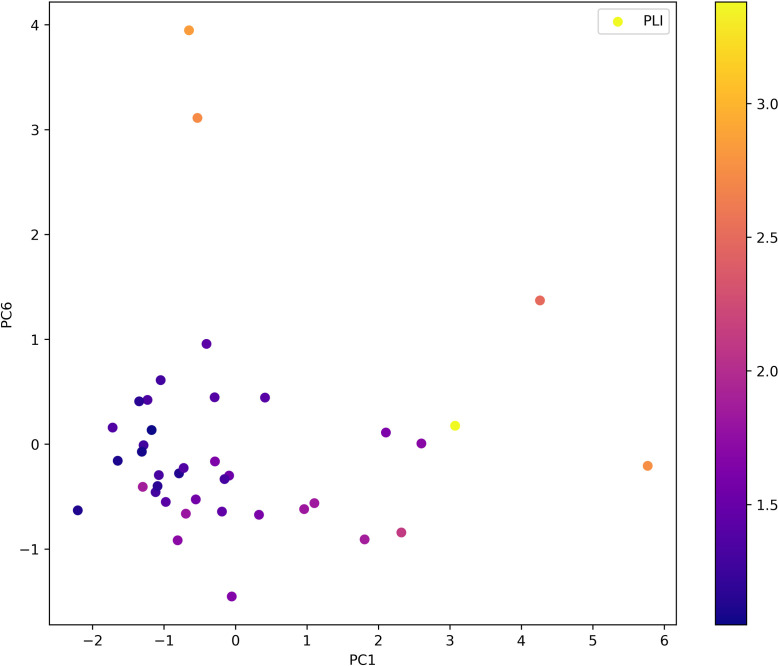
Correlation graph for PC1, PC6, and PLI.

Another notable observation emerges when plotting PC1 and PC6, representing the maximum reflecting environmental indicators PLI and Zc. From Fig 7, we infer that points with higher PLI values are situated at a distance from the cluster of points with lower PLI values and are not clustered together but rather lay separately. The uneven distribution of environmental load indicates that certain areas carry a heavier burden of pollutants than others. The presence and concentration of HMs across the studied territory also display spatial randomness. It is challenging to identify discernible trends in HM concentration distribution within the industrial region’s border due to contamination stemming from numerous enterprises and the intricate routes of distribution. Moreover, contamination’s character exhibits significant variation from one location to another. All of this evidence leads us to conclude that the geological processes in the studied area are insufficient in comparison to technogenic processes. Therefore, the studied area is the result of technogenesis processes.

### Assessment of potential environmental risk

The evaluation of potential environmental risk hinges upon a complex analysis involving various indices that delineate the extent of deviations in HM concentrations from both the maximum permissible thresholds and background concentrations. This assessment encompasses the spatial distribution of these indices, in conjunction with the computation of mean values spanning the entire study area.

The key features of the environmental impact are outlined in Table 3. Based on traditional methods using a PLI parameter, the whole study area is subject to moderate pollution. However, the Zc reveals that soil contamination levels remain permissible, with a Zc value lower than 16. The B indicates insignificant pollution, with a score of 79.52 out of 100. Consequently, an overall assessment gives a rather positive picture of the soil in the industrial region.

**Table 3:** Average environmental impact indicators in the NIZ of Pavlodar.

Element	Background (mg·kg^ − 1^)	Average HQ	Average CF	Min-Max Range CF	Median CF	PLI	Zc	D	Df	B
Fe	20,680	0.51	0.99	0.6-1.7	1.00	1.31	1.66	0.83	0.66	79.52
Mn	600	0.37	0.92	0.4-3.17	0.77					
Cr	440	1.02	0.46	0-1.86	0.34					
Sr	90	0.29	1.93	1.22-2.78	1.89					
Zn	60	2.21	2.02	0.5-15.17	1.17					
Cu	80	1.97	0.81	0-7.88	0					
Pb	32^1^	1.47	1.47	0-6.25	1.56					
Ni	20^1^	1.71	1.71	0-7.5	0					
Mo	50^1^	0.23	0.23	0-8.8	0					
V	150^1^	0.05	0.05	0-0.6	0					
Hg	2.1^1^	1.22	1.22	0-47.6	0					
Co	50^1^	0.04	0.04	0-1.6	0					

^1^Element was not detected in the background soil samples. The MPC value was used for calculations.

Among the elements, Zn and Sr exhibit the highest average CF values at 2.02 and 1.93, respectively, indicating significant contamination relative to their background levels. Pb also shows elevated contamination with an average CF of 1.47, while Fe and Mn have moderate CF values near 1, suggesting contamination close to background levels.

The minimum and maximum CF ranges reveal substantial variability in contamination across the study area. For example, Pb has a wide range from 0 to 6.25, highlighting localized hotspots of severe pollution. Similarly, Zn’s range extends up to 5.17, further emphasizing its significant spatial variability. Median CF values provide a central measure of contamination for each element. Pb has a relatively high median CF of 1.56, confirming its widespread impact. In contrast, elements like Mo, V, and Co have median CF values of 0, indicating minimal contamination in most locations. Overall, the CF analysis suggests that Zn, Sr, and Pb are the most concerning pollutants in terms of both average contamination and spatial variability. According to the average CF level, the metals were ranked in the following order:


Zn> Sr> Ni> Pb> Hg> Fe> Mn> Cu> Cr> Mo > V> Co.

These findings highlight the need for targeted remediation efforts in areas with elevated CF values for these metals.

Moreover, the clustered nature of the spatial distribution of the studied HMs suggests the presence of considerable technogenic anomalies. This is particularly important when considering the fact that the most influential elements fall under the first hazard class, including Zn, Pb, and Hg, and the second hazard class, including Cr, Cu, and Ni. Therefore, it is imperative to investigate the spatial distribution of environmental impact indicators to evaluate the distribution of environmental risk.

**Fig 8:**
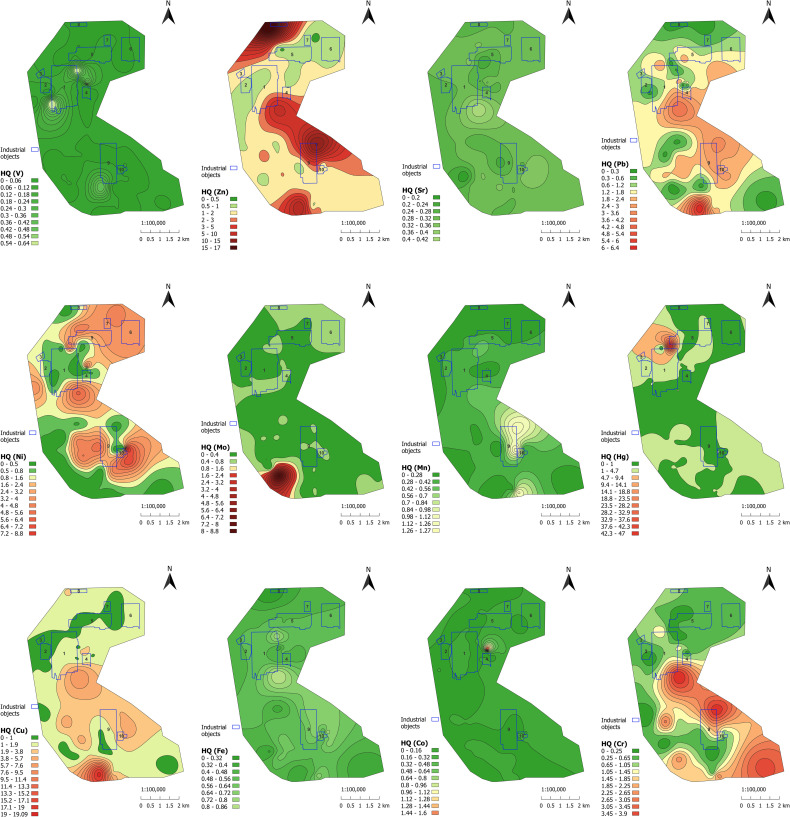
Spatial distribution of HQs. Industrial objects: 1 – Pavlodar petrochemical plant, 2 – Neftechim LTD, 3 – solid waste accumulator, 4 – TPP-3, 5 – former Pavlodar chemical plant (industrial site 1), 6 – former Pavlodar chemical plant (industrial site 2), 7 – calcined petroleum coke production plant, 8 – ponds for mercury waste, 9 – KSP-Steel (former Pavlodar tractor plant), 10 – TPP-2.

Fig 8 shows the spatial distribution of HQ for 12 HMs. The concentrations of Mn, Fe, Sr, Co, and V did not exceed or only slightly exceed the MPC levels, while contamination by Hg and Mo was strongly localized. The distribution of Cr, Ni, and Pb exhibited cluster characteristics. Exceedances of the MPC for Zn and Cu were observed throughout nearly the entire research area.

It is characteristic that chromium, zinc, copper, nickel, and lead exhibit a similar spatial distribution trend. The highest concentrations are located between two TPPs and extend in a southeastern direction towards Pavlodar city. Excessive amounts of zinc, nickel, and mercury were found in the area of the former Pavlodar chemical plant, which used large quantities of mercury as a liquid cathode in the production of chlorine and caustic soda. Extremely high levels of zinc, copper, lead, molybdenum, and chromium were detected in the southern region of the KSP-Steel enterprise (previously known as Pavlodar tractor plant), which is involved in metal processing. The abnormalities found could be attributed to dust emissions from the industrial processes of metal treatment.

Thus, visualization of the collected data reveals the highest levels of pollution caused by various contaminants in different locations. This assists in objectively identifying the most significant pollutants and their distribution patterns.

**Fig 9:**
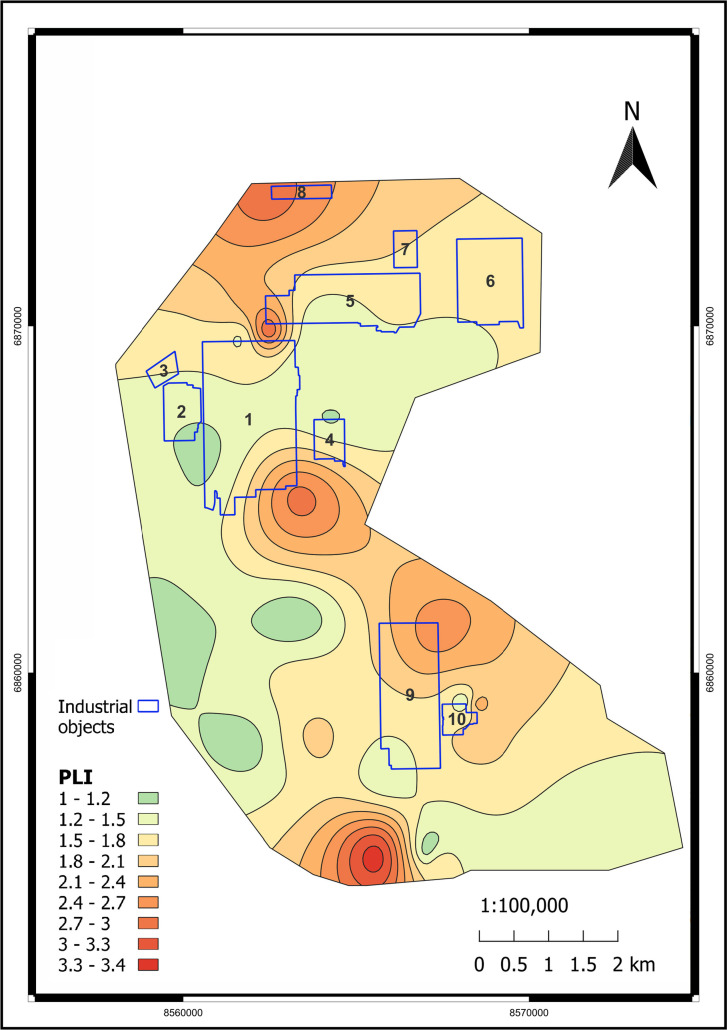
PLI spatial distribution in the study area. (Interpolation: SAGA 7.8.2 multilevel b-spline in QGIS v. 3.28.11. [50]). Industrial objects: 1 – Pavlodar petrochemical plant, 2 – Neftechim LTD, 3 – solid waste accumulator, 4 – TPP-3, 5 – former Pavlodar chemical plant (industrial site 1), 6 – former Pavlodar chemical plant (industrial site 2), 7 – calcined petroleum coke production plant, 8 – ponds for mercury waste, 9 – KSP-Steel (former Pavlodar tractor plant), 10 – TPP-2.

**Fig 10:**
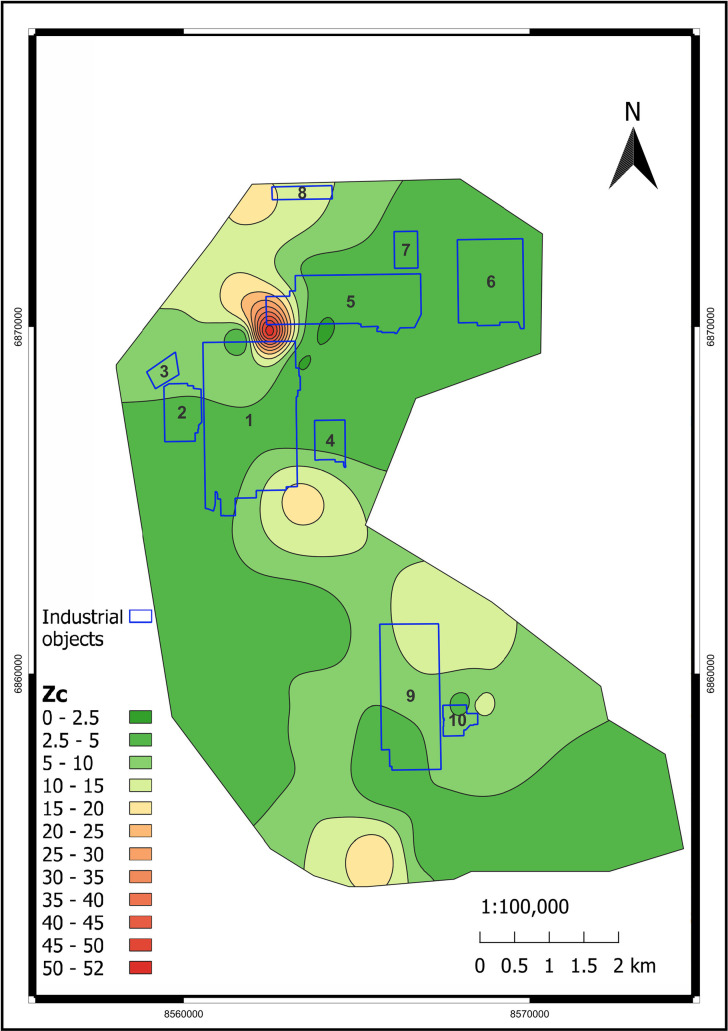
Zc spatial distribution in the study area. (Interpolation: SAGA 7.8.2 multilevel b-spline in QGIS v. 3.28.11. [50]). Industrial objects: 1 – Pavlodar petrochemical plant, 2 – Neftechim LTD, 3 – solid waste accumulator, 4 – TPP-3, 5 – former Pavlodar chemical plant (industrial site 1), 6 – former Pavlodar chemical plant (industrial site 2), 7 – calcined petroleum coke production plant, 8 – ponds for mercury waste, 9 – KSP-Steel (former Pavlodar tractor plant), 10 – TPP-2.

**Fig 11:**
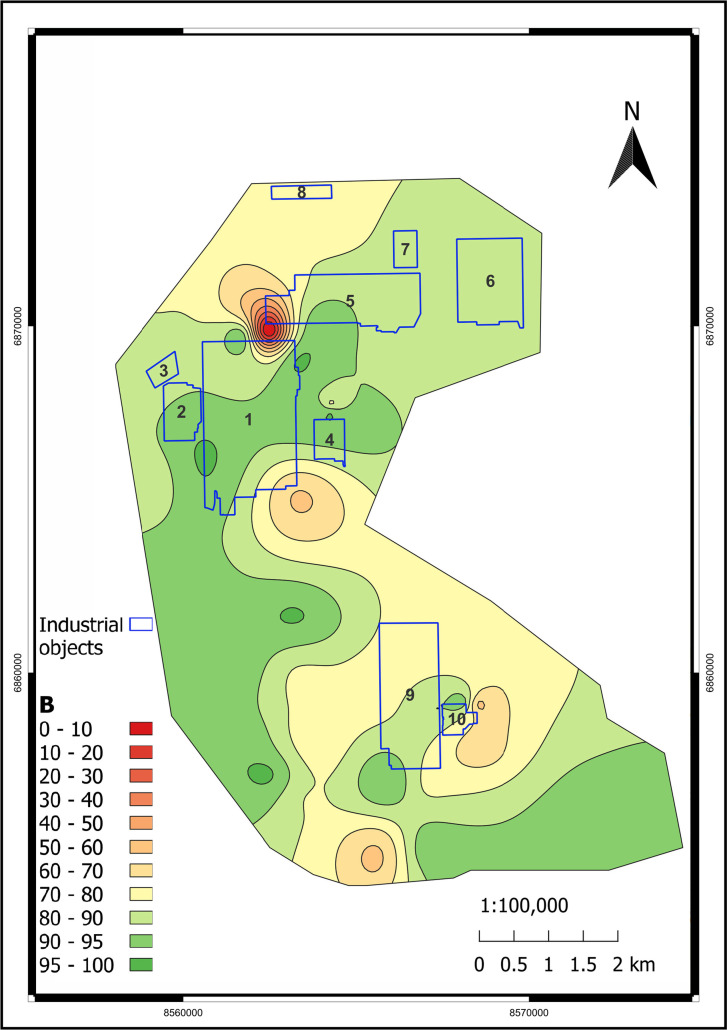
B spatial distribution in the study area. (Interpolation: SAGA 7.8.2 multilevel b-spline in QGIS v. 3.28.11. [50]). Industrial objects: 1 – Pavlodar petrochemical plant, 2 – Neftechim LTD, 3 – solid waste accumulator, 4 – TPP-3, 5 – former Pavlodar chemical plant (industrial site 1), 6 – former Pavlodar chemical plant (industrial site 2), 7 – calcined petroleum coke production plant, 8 – ponds for mercury waste, 9 – KSP-Steel (former Pavlodar tractor plant), 10 – TPP-2.

Figs 9–11 illustrate the spatial distribution of environmental indicators - PLI, Zc, and B. The majority of the territory can be characterized as moderately contaminated based on PLI and B indicators. Regarding toxicity, the Zc indicator is more informative, and we can observe that the vast majority of the study area is within the permissible range. Nevertheless, graphical visualization assists in identifying a heightened level of environmental risk within the area of the former Pavlodar chemical plant. The Zc indicator-based toxic classification for that location is identified as hazardous. Such a conclusion is supported by the B, which was as low as 3 in that area. Based on the PLI analysis, the most contaminated areas are located near the former Pavlodar petrochemical plant (industrial site 1), which extends to the mercury waste ponds. Additionally, the second highly contaminated area is situated between TPP-3 and the KSP-Steel and TPP-2 enterprises. The third area is located below KSP Steel and has the highest level of PLI. This site is significantly polluted with toxic HMs.

Thus, spatial visualization aids in a more precise assessment of environmental risk and identification of the areas more susceptible to contamination.

We also employed graphical data to calculate spatial shares of various hazardous classes within the study area, based on classifications by Zc (Table 4) and PLI (Table 5). From the spatial distribution of the Zc indicator, it is determined that although 96% of the study area has a permissible contamination level, there are still 3.2% moderately dangerous and 0.395% dangerous areas. Using PLI, we also determined that 93.7% of the territory has moderate contamination and 0.7% of the territory has considerable contamination.

**Table 4:** Spatial shares of soil at various contamination levels in the study area using the Zc indicator.

Soil contamination level (Zc)	Area, m^2^	Share, %
Permissible	194,969,101	96.387
Moderately hazardous	6,508,727	3.218
Hazardous	799,578	0.395
Extremely hazardous	–	–

**Table 5:** Spatial shares of soil at various contamination levels in the study area using the PLI.

Soil contamination level (PLI)	Area, m^2^	Share, %
No contamination	–	–
Low contamination	11,208,294	5.541
Moderate contamination	189,648,493	93.757
Considerable contamination	1,420,620	0.702
Very high contamination	–	–

Thus, in assessing environmental risk, it is reasonable to not solely rely on average indicator values but also conduct spatial analysis of integrated environmental indices such as PLI or Zc. By implementing these strategies, we can accurately identify locations with heightened environmental risk and investigate trends and causes of technogenic anomalies [66].

## Discussion

The obtained results are consistent with numerous other studies investigating soil conditions in industrial zones. So, Wang et al. [67] studied Industrial Parks in the West of Shaanxi Province, China, Mohammadi et al. [68] - Industrial Zone in Neyshabur, Iran, Yaylali-Abanuz [69] - Gebze industrial area, Turkey. All of these studies concluded that Industrial Zones are the harder-hit areas of HM pollution in soil and the intensification of human activities, the growing industrialization, and the rapid urbanization largely influenced the concentration levels of HMs.

Industrialization has undoubtedly been a significant driver of economic growth and societal advancement. However, it is important to acknowledge that it also has significant environmental implications, particularly in urban industrial zones. The rapid increase in demand for natural resources as raw materials for manufacturing can lead to exploitative behavior, which in turn can deplete natural resources and compromise environmental sustainability [70]. Industrial processes, such as mining, fossil fuel burning, and industrial waste, have been identified as significant contributors to environmental pollution [1].

The main enterprises of the northern industrial zone of Pavlodar are the Pavlodar Petrochemical Plant, TPP-2, TPP-3, “KSP Steel, JSC “Caustic”, and “Neftechim LTD”. According to published data, heavy metals are among the primary pollutants in air emissions. Air emissions are the main source of soil pollution, particularly affecting its surface layer. A recent article [71] published information on the quantity of air emissions containing heavy metals from the major enterprises in the northern industrial zone of Pavlodar.

Table 6 demonstrates the pollution load from various industries, revealing significant variability in the types and quantities of pollutants emitted. The primary contributors to pollution are inorganic dust and petrol emissions, with the highest total emissions originating from TPP-3 (4888.68 tons/year) and the KSP Steel plant (350.687 tons/year). These industries predominantly release particulate matter (inorganic and abrasive dust), metals (iron, manganese, aluminum), and chemical compounds (chlorine, nickel oxide). Among the industries listed, TPP-3 stands out as the largest polluter due to its high emissions of inorganic dust. Additionally, KSP Steel is notable for its release of various metals and petrol.

**Table 6:** Pollutant emissions from industrial facilities in the NIZ of Pavlodar.

Name of pollutant	Pavlodar petrochemical plant (kg/year)	TPP-2 (kg/year)	TPP-3 (kg/year)	KSP-Steel (kg/year)	Casting LLP (kg/year)	Neftechim LTD (kg/year)	JSC Caustic (kg/year)
Iron	1.1	5.2	2176.3	16,915.4	1450.8	6	265.01
Manganese and its compounds	0.1	11.5	50.6	18,000.84	5.29	1	5.56
Aluminum	78,877.7			1322.7			0.0037
Chromium	0.1	0.3	1.46	6	0.04		0.03
Chlorine							175.2
Nickel oxide	0.005	0.2	0.01	0.0003	0.0039		0.0029
Lead		0.02		0.002			0.0004
Copper		1.7	20.92	0.05			
Zinc		0.03		313.5			
Petrol	48,740.9		50.77	35.2			675.9
Kerosene		5.6		14.2	81.5		
Inorganic dust	188,492	1,177,692	4,885,213	304,247.2	220,000	790	1436.55
Abrasive dust	1.1	65.42	150.66	3080.8	33.36	1	209.72
Wood dust		76.71	563.8	6741.1			191.46
Rubber dust				10.3			
Metal dust		165.68	456.1				
Sum	316,113.005	1,178,024.36	4,888,683.62	350,687.2923	221,570.9939	798	2959.437

Note: This table is reproduced from [71], originally published under CC BY-NC-ND 4.0 License.

The data obtained from soil analysis correlate with information on air emissions from major enterprises in the industrial zone under study. For example, elevated levels of zinc, chromium, manganese, and iron have been observed near KSP Steel. Similarly, increased concentrations of iron, manganese, and chromium were found in areas close to TPP-3 and TPP-2.

Thus, considering the composition of emissions from the main enterprises of the northern industrial zone of Pavlodar, it can be concluded that they are the primary source of elevated heavy metal levels in the soil within the studied area.

The results of the study on heavy metal content in the soil align with findings from research on heavy metal concentrations in the snow cover of the northern industrial zone of Pavlodar [71]. High concentrations of elements such as chromium (Cr), zinc (Zn), lead (Pb), and manganese (Mn) were detected, indicating significant anthropogenic impact. Chromium concentrations reached 820 mg/kg in the soil and 316.6 mg/kg in the snow cover, exceeding permissible concentration limits. Both studies demonstrate a clustered distribution of pollutants, with the highest concentrations of heavy metals found near major industrial facilities, such as the Pavlodar Petrochemical Plant and TPP-3.

Wind direction plays a crucial role in the dispersion of pollutants. Researchers studying the snow cover note that southern and southwesterly winds contribute to the spread of pollutants northeastward from industrial sites.

In Table 7, comparative information is provided on the content of certain heavy metals in the soil and snow cover of the northern industrial zone of Pavlodar.

**Table 7:** Comparison of Heavy Metal Concentrations in Soil and Snow Cover in the Northern Industrial Zone of Pavlodar.

HM	Mean concentration in soil (mg/kg)	Mean concentration in snow cover (mg/kg) [71]
Cr	203.85	316.6
Zn	121.28	286.2
Pb	46.92	158.5
Mn	553.85	638.6

The concentration of heavy metals in snow is often higher than in soil due to differences in accumulation and analysis processes. Snow acts as a direct deposition medium, capturing pollutants from the atmosphere, including emissions from industrial activities, vehicles, and other sources. These pollutants settle on the snow surface and are not subject to the same leaching or absorption processes that affect the soil. Additionally, snow analysis typically involves filtering solid particulates from meltwater, and isolating heavy metals without interference from the soil’s organic and mineral components. This results in a more concentrated measurement of metals in snow compared to soil. For instance, in Pavlodar’s northern industrial zone, chromium concentrations reached 316.6 mg/kg in snow but averaged 203.85 mg/kg in soil, highlighting this difference.

In the future, several key trends are likely to influence the global landscape of industrialization. These include digitalization, production rebalancing, and industrial greening [72]. It is important for countries to take environmental factors into account when designing recovery strategies [73].

Environmental risk assessment is a critical process used to estimate the probability of an adverse outcome of environmental changes caused by human activities. It follows the scientific process of hazard determination and the health risks associated with exposure to contamination. The result of the evaluation helps determine ways to remediate or remove environmental stressors and prevent or mitigate possible ecological damage [74]. Environmental risk assessment and sustainable development are completely interconnected concepts because practicing the former is a commitment to affirm the latter [75].

HMs have been detected not only in Industrial Zones but also in urban road dust from various cities across different continents, indicating a widespread problem [76]. The effects of HM contamination on human health are also significant. Exposure to HMs can lead to chronic and acute toxicity, which can cause retardation, neurotoxicity, kidney damage, the development of different cancers, liver and lung damage, and even death in case of a huge amount of exposure [77]. Specific HMs, such as mercury, lead, and cadmium, are of greatest concern because of their ability to travel long distances in the atmosphere [78]. These metals often contribute the most to pollution load indices due to their toxicity. This is consistent with our results and previous research.

Elevated concentrations of heavy metals such as zinc (Zn), strontium (Sr), nickel (Ni), lead (Pb), and mercury (Hg) pose significant risks to human health, especially in areas near industrial facilities or regions with high contamination levels. These metals, when exceeding permissible thresholds, can have toxic effects on various physiological systems, leading to both acute and chronic health outcomes.

Zinc, while an essential trace element necessary for enzymatic functions and immune responses, becomes toxic at elevated levels. Excessive zinc can interfere with the absorption of other critical metals like copper and iron, potentially causing immune dysfunction, gastrointestinal distress, and neurological issues [79,80].

Strontium, commonly found in contaminated soils and water near industrial sites, can replace calcium in bones when ingested in high amounts. This substitution weakens bone structure and increases the risk of skeletal abnormalities. The long-term implications of strontium exposure are particularly concerning for children and pregnant women due to their higher calcium demands [81,82].

Nickel is another metal that poses health risks when present in high concentrations. Chronic exposure to nickel is associated with allergic reactions, respiratory issues, and even carcinogenic effects due to its ability to damage DNA. Industrial emissions are a major source of nickel contamination, making populations near such facilities particularly vulnerable [81,83].

Lead is a well-documented neurotoxin that can cause severe health issues even at low exposure levels. It primarily affects the central nervous system and is especially harmful to children, where it can lead to developmental delays, reduced IQ, and behavioral problems. In adults, lead exposure is linked to hypertension, kidney damage, and reproductive issues. Its persistence in the environment means that contaminated soils and water sources can remain hazardous for decades [82,84].

Mercury is one of the most toxic heavy metals with widespread effects on human health. It primarily targets the nervous system but also affects the kidneys and immune system. Chronic mercury exposure can result in cognitive impairments, motor dysfunctions, and cardiovascular problems. The bioaccumulation of mercury in aquatic ecosystems further exacerbates its impact on human health through the consumption of contaminated fish [84,85].

The mechanisms by which these heavy metals exert their toxic effects often involve oxidative stress, enzyme inhibition, and disruption of cellular processes such as DNA repair and apoptosis. These disruptions can lead to chronic illnesses including cancers, cardiovascular diseases, neurodegenerative disorders like Parkinson’s or dementia, and immune system dysfunctions [84].

Populations living near industrial facilities or areas with significant contamination are at heightened risk due to prolonged exposure through ingestion (contaminated food or water), inhalation (airborne particles), or dermal contact. Children are particularly vulnerable because of their developing bodies and higher rates of absorption relative to body weight [79,80].

From an environmental and public health perspective, these risks underscore the urgent need for mitigation strategies. These include stricter industrial regulations to limit heavy metal emissions, regular monitoring of soil and water quality in affected areas, public awareness campaigns about safe practices to reduce exposure, and remediation efforts such as soil decontamination or water filtration systems. The economic consequences of HM contamination are also substantial. The contamination can lead to a decline in agricultural productivity due to soil and crop contamination [86]. To the south of the studied area in the NIZ of Pavlodar, there are personal agricultural sites belonging to Pavlodar citizens. These sites are affected by the negative impact of air transportation of HMs. Moreover, the cost of remediation strategies to remove these pollutants from the environment can be high [87]. Therefore, HM pollution not only poses environmental and health risks but also has significant economic implications.

The spatial distribution of HMs in industrial regions is a critical aspect of environmental risk assessment. Identifying hotspots of HM contamination is a significant challenge due to the complex nature of industrial pollution. The contamination levels of HMs can vary extensively among cities, countries, continents, and periods [76]. Moreover, the co-contamination of HMs is widespread in industrial areas due to the presence of HMs in industrial waste [88]. For instance, in our research area, the concentration coefficients for each metal and pollution indices are unevenly distributed, displaying a pronounced cluster character of pollutant distribution. Determining trends is difficult due to the large number of plants located in this area. However, it is possible to identify that the maxima of the PLI are mainly located to the southwest of the TPPs and at some distance from their territories. This corresponds to the average wind rose for Pavlodar city. As well that agrees with the theory of distribution of smoke-heavy components that are transported by air [89]. Moreover, there is another location of high PLI in the northern part of the studied area near the Hg accumulating ponds. The high PLI level there is generally due to high Hg concentration. Although only three locations have Hg concentrations above the MPC, the extreme toxicity of this element results in a wide zone of contamination.

Sustainable development in industrial regions is a critical aspect of modern economic growth and environmental stewardship. It involves the creation of resilient infrastructure, the promotion of sustainable industrialization, and the fostering of innovation. Informed decision-making plays a crucial role in this process, as it enables development to become more sustainable and resilient [90]. It pushes development decision-makers to understand and acknowledge that all development choices involve the creation of uncertain risks, as well as opportunities [90].

The promotion of environmental quality and human health in rapidly expanding industrial areas is a key component of sustainable development. Industrial pollution has severe toxicological effects on the environment and human health [70,91]. Therefore, minimization of industrial pollution should be the topmost policy agenda in the industrialized countries [70]. Occupational health is an area of work in public health to promote and maintain the highest degree of physical, mental, and social well-being of workers in all occupations [92]. Protecting workers’ health is a fundamental aspect of promoting environmental quality and human health in industrial areas [93].

The broader context of research gaps and the need to develop future research directions lies in the limitations of the existing literature, in particular the lack of up-to-date monitoring and comprehensive analysis of environmental risks in industrial areas. Current statistics indicate an increase in respiratory diseases and cancer cases in industrial regions, emphasizing the urgent need for more recent and detailed studies to address these environmental issues. The study area requires targeted remediation strategies focusing on hotspots near industrial facilities, particularly around the former chemical plant, mercury waste ponds, and KSP-Steel territory. Specific measures should include soil washing techniques for Zn and Pb-contaminated areas, phytoremediation using metal-accumulating plants, and chemical stabilization methods for mercury-contaminated zones. Industrial sites showing high PLI values (above 2.0) and dangerous Zc levels require immediate intervention through containment and treatment of contaminated soils.

Environmental monitoring should be implemented through a comprehensive system including

- regular soil sampling and analysis in identified high-risk zones, particularly areas with PLI values exceeding 1.5;
- installation of continuous air quality monitoring stations near major industrial facilities to track metal-containing emissions;
- implementation of groundwater monitoring wells to detect potential leaching of heavy metals;
- development of a GIS-based database to track temporal changes in contamination levels and effectiveness of remediation efforts.

These recommendations aim to reduce environmental risks while promoting sustainable industrial development in the region.

Despite its contributions, this study has several limitations. The analysis relies primarily on surface soil data, which may not fully capture the vertical migration dynamics of heavy metals or their seasonal variations. The sampling was conducted during a specific period (September-October 2022), limiting understanding of temporal changes in contamination levels. The number of sampling points (39) could be increased to provide more detailed spatial coverage of the study area, and a more structured sampling grid could be implemented to ensure systematic coverage of the territory. Additionally, the study focused on total metal concentrations without examining their bioavailability or speciation forms, which are crucial for accurate risk assessment.

## Conclusion

The purpose of this study is to assess the spatial distribution of HMs, including Fe, Mn, Cr, Sr, Zn, Cu, Pb, Ni, Mo, V, Hg, and Co, in soils of the NIZ of Pavlodar, Kazakhstan, and to evaluate the associated environmental risks. Given the region’s significance as a major industrial hub, this research aims to address the lack of recent and detailed studies on soil contamination in rapidly evolving industrial areas. The study employed a combination of environmental risk indices (PLI, Zc, HQ), statistical tools (PCA, correlation analysis), and GIS-based spatial visualization to assess HM contamination in soils. This integrated approach identified pollution hotspots, linked contamination to industrial activities, and provided a replicable framework for environmental monitoring and risk management.

The study revealed significant spatial variability in HM concentrations in the soils in the studied area. The most prevalent elements were Fe, Mn, and Cr, with average concentrations of 20,406.92 mg/kg, 553.85 mg/kg, and 203.85 mg/kg, respectively. Zn (121.28 mg/kg) and Sr (173.33 mg/kg) also exhibited high average concentrations. Exceedances of the MPCs were observed for Zn, Pb, Cu, Cr, and Ni in 79%, 67%, 46%, 79%, and 36% of sampling points, respectively.

The PLI and the Zc demonstrated moderate to locally significant contamination levels. PLI values ranged from 1.05 to 3.4, with 93.757% of the area classified as moderately polluted and 0.702% significantly polluted. The analysis of Zc revealed permissible contamination levels for 96.387% of the area, with 3.218% classified as moderately hazardous and 0.395% as hazardous. The highest index values were recorded near key industrial facilities, such as mercury waste ponds and the KSP-Steel production site.

CFs indicated significant relative pollution by Zn (2.02), Sr (1.93), and Pb (1.47). HQs for Zn and Cu exceeded safe thresholds across most sites, particularly near industrial hotspots. Correlations between elements, such as Fe-Mn (r = 0.61) and Sr-Ni (r = 0.55), highlighted common sources or distribution mechanisms.

The spatial distribution of pollution in the NIZ of Pavlodar reflects unique and heterogeneous patterns influenced by industrial activities. The analysis of heavy metal concentrations revealed clustered contamination zones, particularly near key industrial facilities such as TPP-2, TPP-3, the former Pavlodar Chemical Plant, KSP-Steel, and mercury waste ponds. These areas exhibited significantly elevated levels of Zn, Pb, Hg, Cu, and Cr, with localized hotspots often surpassing MPCs. Technogenic factors, such as emissions, industrial waste disposal, and wind direction, were identified as primary drivers of pollutant patterns.

The PLI and Zc showed uneven spatial contamination, with distinct clusters near industrial facilities and a gradual decrease in pollution levels with distance from emission sources. The study also observed a southeast contamination trend in alignment with wind patterns. Notably, a high PLI area was identified in the northern region near mercury storage ponds, where Hg contamination created a wide pollution zone despite limited sampling points exceeding Hg’s MPC due to its extreme toxicity.

The results underline the anthropogenic origin of contamination, driven by industrial activity. Elevated levels of highly toxic elements, including Hg, Pb, and Zn, represent significant ecological and health risks. Targeted monitoring and remediation strategies are essential for managing contaminated areas and mitigating environmental impacts.

The elevated concentrations of Zn, Sr, Ni, Pb, and Hg pose serious threats to human health by disrupting critical biological processes and increasing the risk of chronic diseases. Addressing these risks requires coordinated efforts between governments, industries, researchers, and communities to safeguard public health while ensuring sustainable environmental practices.

## Supporting information

S1 Table Geochemical characterization of soil samples. (DOCX)
