# Supplementary material for: Environmental risk assessment for sustainable industrial urban development: The case of northern industrial zone of Pavlodar, Kazakhstan
Source: PLoS One. 2025 Apr 16;20(4):e0320835. doi: 10.1371/journal.pone.0320835 (PMC12002492; doi:10.1371/journal.pone.0320835)
Supplement: S1 Table — (DOCX) [file pone.0320835.s001.docx]

Table S1. Geochemical characterization of soil samples.

| **Sample ID** | **Cr**  **(mg·kg^-1^)** | **Mn**  **(mg·kg^-1^)** | **Fe**  **(mg·kg^-1^)** | **Zn**  **(mg·kg^-1^)** | **Sr**  **(mg·kg^-1^)** | **Co**  **(mg·kg^-1^)** | **Cu**  **(mg·kg^-1^)** | **Hg**  **(****mg·kg^-1^)** | **Ni**  **(mg·kg^-1^)** | **Pb**  **(mg·kg^-1^)** | **V**  **(mg·kg^-1^)** | **Mo**  **(mg·kg^-1^)** |
| --- | --- | --- | --- | --- | --- | --- | --- | --- | --- | --- | --- | --- |
| 1 | 330 | 240 | 13570 | 380 | 170 | n.d. | 630 | n.d. | n.d. | 200 | n.d. | n.d. |
| 2 | 460 | 540 | 22870 | 70 | 180 | n.d. | n.d. | n.d. | n.d. | n.d. | n.d. | 440 |
| 3 | 530 | 530 | 19190 | 130 | 180 | n.d. | 150 | n.d. | n.d. | n.d. | n.d. | n.d. |
| 4 | 310 | 460 | 15580 | 70 | 150 | n.d. | 90 | n.d. | n.d. | n.d. | n.d. | n.d. |
| 5 | 100 | 470 | 17630 | 60 | 160 | n.d. | 90 | n.d. | n.d. | 50 | n.d. | n.d. |
| 6 | 140 | 320 | 19810 | 90 | 150 | n.d. | 90 | n.d. | n.d. | n.d. | n.d. | n.d. |
| 7 | 820 | 770 | 34360 | 290 | 250 | n.d. | 230 | n.d. | 110 | 130 | n.d. | n.d. |
| 8 | 140 | 350 | 20710 | 50 | 190 | 80 | 70 | n.d. | 80 | 40 | 60 | n.d. |
| 9 | 240 | 410 | 14650 | 70 | 170 | n.d. | n.d. | n.d. | n.d. | 70 | n.d. | n.d. |
| 10 | n.d. | 280 | 18460 | 30 | 190 | n.d. | n.d. | n.d. | 90 | 40 | n.d. | n.d. |
| 11 | 190 | 370 | 21390 | 60 | 180 | n.d. | 90 | n.d. | 80 | 60 | n.d. | n.d. |
| 12 | n.d. | 710 | 29810 | 70 | 150 | n.d. | n.d. | n.d. | n.d. | n.d. | 90 | n.d. |
| 13 | 240 | 390 | 16310 | 110 | 180 | n.d. | n.d. | n.d. | n.d. | 80 | n.d. | n.d. |
| 14 | 160 | 320 | 11380 | 910 | 160 | n.d. | 60 | n.d. | n.d. | n.d. | n.d. | n.d. |
| 15 | n.d. | 430 | 18390 | 40 | 170 | n.d. | 90 | n.d. | n.d. | n.d. | n.d. | n.d. |
| 16 | n.d. | 490 | 21210 | 80 | 200 | n.d. | n.d. | 100 | 90 | n.d. | n.d. | n.d. |
| 17 | 330 | 1900 | 27580 | 140 | 180 | n.d. | 80 | n.d. | n.d. | 90 | n.d. | n.d. |
| 18 | 150 | 370 | 16390 | 70 | 160 | n.d. | n.d. | n.d. | n.d. | 60 | n.d. | n.d. |
| 19 | 100 | 300 | 17690 | 30 | 170 | n.d. | n.d. | n.d. | 80 | n.d. | n.d. | n.d. |
| 20 | 80 | 690 | 20270 | 70 | 130 | n.d. | n.d. | n.d. | n.d. | 60 | n.d. | n.d. |
| 21 | n.d. | 460 | 19780 | 40 | 160 | n.d. | n.d. | n.d. | n.d. | n.d. | n.d. | n.d. |
| 22 | n.d. | 350 | 17180 | 50 | 110 | n.d. | n.d. | n.d. | n.d. | 60 | n.d. | n.d. |
| 23 | 90 | 480 | 20040 | 40 | 170 | n.d. | n.d. | n.d. | n.d. | 40 | 90 | n.d. |
| 24 | 260 | 420 | 19860 | 80 | 180 | n.d. | n.d. | n.d. | n.d. | 70 | n.d. | n.d. |
| 25 | 190 | 310 | 13190 | 60 | 170 | n.d. | n.d. | n.d. | n.d. | n.d. | n.d. | n.d. |
| 26 | 130 | 330 | 20380 | 60 | 140 | n.d. | n.d. | n.d. | 100 | 60 | n.d. | n.d. |
| 27 | 80 | 500 | 21480 | 90 | 190 | n.d. | n.d. | n.d. | n.d. | n.d. | 60 | n.d. |
| 28 | 100 | 660 | 20960 | 100 | 190 | n.d. | 110 | n.d. | 100 | 80 | n.d. | n.d. |
| 29 | 780 | 1760 | 28230 | 530 | 180 | n.d. | 120 | n.d. | n.d. | 100 | n.d. | n.d. |
| 30 | 150 | 560 | 26550 | 90 | 210 | n.d. | 90 | n.d. | 140 | 80 | n.d. | n.d. |
| 31 | n.d. | 470 | 20250 | 60 | 160 | n.d. | n.d. | n.d. | n.d. | 50 | n.d. | n.d. |
| 32 | 180 | 580 | 19480 | 60 | 160 | n.d. | 130 | n.d. | n.d. | 60 | n.d. | n.d. |
| 33 | 90 | 440 | 20830 | 60 | 180 | n.d. | n.d. | n.d. | 70 | 60 | n.d. | n.d. |
| 34 | 200 | 540 | 29240 | 120 | 210 | n.d. | 160 | n.d. | 150 | 70 | n.d. | n.d. |
| 35 | n.d. | 520 | 19740 | 90 | 170 | n.d. | n.d. | n.d. | 80 | 50 | n.d. | n.d. |
| 36 | 120 | 600 | 23270 | 110 | 190 | n.d. | 140 | n.d. | 90 | 70 | n.d. | n.d. |
| 37 | 150 | 330 | 17520 | 50 | 160 | n.d. | n.d. | n.d. | 70 | 40 | n.d. | n.d. |
| 38 | 420 | 1570 | 25700 | 120 | 180 | n.d. | 110 | n.d. | n.d. | 60 | n.d. | n.d. |
| 39 | 690 | 380 | 14940 | 100 | 180 | n.d. | n.d. | n.d. | n.d. | n.d. | n.d. | n.d. |

^*^ n.d. – not detected
